# Comparative evaluation of a laboratory-developed real-time PCR assay and RealStar® Adenovirus PCR Kit for quantitative detection of human adenovirus

**DOI:** 10.1186/s12985-018-1059-7

**Published:** 2018-09-27

**Authors:** Samson S. Y. Wong, Cyril C. Y. Yip, Siddharth Sridhar, Kit-Hang Leung, Andrew K. W. Cheng, Ami M. Y. Fung, Ho-Yin Lam, Kwok-Hung Chan, Jasper F. W. Chan, Vincent C. C. Cheng, Bone S. F. Tang, Kwok-Yung Yuen

**Affiliations:** 1 Department of Microbiology, The University of Hong Kong, Hong Kong, China; 2 State Key Laboratory of Emerging Infectious Diseases, The University of Hong Kong, Hong Kong, China; 3 Research Centre of Infection and Immunology, The University of Hong Kong, Hong Kong, China; 4 Carol Yu Centre for Infection, The University of Hong Kong, Hong Kong, China; 5 Department of Microbiology, Queen Mary Hospital, Hong Kong, China; 6 Department of Pathology, Hong Kong Sanatorium and Hospital, Hong Kong, China; 7 The Collaborative Innovation Center for Diagnosis and Treatment of Infectious Diseases, The University of Hong Kong, Hong Kong, China

**Keywords:** Human adenovirus, Real-time PCR, RealStar® Adenovirus PCR Kit

## Abstract

**Background:**

Human adenoviruses are common causes of community-acquired respiratory tract and enteric infections. Severe disseminated infections with high mortality rates may be seen in immunocompromised individuals. An accurate and cost-effective quantitative assay is essential not only for laboratory diagnosis of adenoviral infections, but also for monitoring of response to antiviral treatment. The diagnostic performance of an in-house quantitative polymerase chain reaction assay was compared to a commercial system.

**Methods:**

The analytical sensitivity, specificity, linearity, precision and accuracy of an in-house adenovirus quantitative polymerase chain reaction assay were evaluated against the RealStar® Adenovirus PCR Kit (Altona Diagnostics GmbH, Hamburg, Germany), using 122 clinical specimens and 18 proficiency testing samples.

**Results:**

Linear regression analysis of the quantitative results by the in-house assay showed the dynamic range from 2.60 to 9 log_10_ (plasma) and 2.94 to 9 log_10_ (viral transport medium) copies/mL, with the coefficient of determination (R^2^) of 0.996 and 0.998, respectively. A dilution series demonstrated the limits of detection and lower limits of quantification for plasma were 2.06 log_10_ and 2.60 log_10_ copies/mL and those for viral transport medium were 2.31 log_10_ and 2.94 log_10_ copies/mL respectively. The precision of the in-house assay was highly reproducible among runs with coefficients of variance ranging from 0.07 to 3.21% for plasma and 0.17% to 2.11% for viral transport medium. A comparison of 52 matched samples showed an excellent correlation between the quantitative viral loads measured by the in-house assay and the RealStar® Adenovirus PCR Kit (R^2^ = 0.984), with an average bias of − 0.16 log_10_ copies/mL.

**Conclusions:**

The in-house adenovirus assay is a sensitive and reliable assay with lower cost for the detection and quantification of adenoviral DNA when compared to the RealStar® Adenovirus PCR Kit.

**Electronic supplementary material:**

The online version of this article (10.1186/s12985-018-1059-7) contains supplementary material, which is available to authorized users.

## Background

Human adenoviruses (HAdV) are common causes of community-acquired infections. In immunocompetent individuals, adenovirus infections often manifest as upper respiratory tract infections, pharyngoconjunctival fever, or diarrhoea. As in the case of other viral respiratory pathogens, community outbreaks of adenovirus infections may occur, especially with respiratory tract infections and keratoconjunctivitis. Such outbreaks may carry substantial morbidity and mortality, particularly in terms of severe respiratory tract involvement [1, 2]. In immunocompromised hosts, adenovirus infections may present as respiratory tract infection, hepatitis, enteritis, haemorrhagic cystitis, disseminated infections, and graft loss in organ transplant recipients. High risk individuals include patients with primary immunodeficiencies (especially severe combined immunodeficiency syndrome), allogenic haematopoietic stem cell transplant and solid organ transplant recipients. Disseminated disease in immunocompromised patients may carry a case-fatality ratio of more than 50% [3].

Currently there are seven species of HAdV (A to G) with over 50 serotypes [3]. Certain species/serotypes are characteristically associated with organ-specific infections. For example, adenoviral keratoconjunctivitis is often associated with species D, while enteric infections are often caused by F40 and F41 [3]. Respiratory tract infections, one of the commonest manifestations of HAdV infections, is frequently due to species B and C. There are considerable geographical variations in the prevalence of various species and serotypes causing respiratory tract infections. In recent studies from China, Malaysia, Croatia, and the USA, for example, species C is often the commonest serotype involved in community-acquired respiratory tract infections, followed by species B; while the serotypes C1, C2, and B3 were most frequently detected [4–8]. Similarly, species C is one of the commonest species involved in infections among the immunocompromised patients [3]. Understanding the epidemiology and prevalence of various HAdV species and serotype (ideally within one’s locality) is important in order to choose the most appropriate diagnostic modality which demonstrates good sensitivity and specificity towards the clinically important strains.

Conventional viral culture has largely been supplanted by nucleic acid amplification tests in the diagnosis of adenovirus infections. The detection of adenoviral DNA by nucleic acid amplification offers excellent sensitivity and shorter turnaround time than viral culture, and is also a powerful tool in the discovery of novel viruses [9]. Nucleic acid amplification assays also allows easier determination of viral serotypes and quantification of viral load in clinical specimens. The determination of viral load is especially important for immunocompromised hosts who have a higher risk of developing severe and disseminated infections. It is now well established that high levels of HAdV DNAemia portends disseminated diseases and monitoring of viral load in peripheral blood and stool in susceptible hosts may have a role in early diagnosis and pre-emptive treatment of high risk individuals [10–14]. Viral load study is also useful in monitoring the response to antiviral therapy such as cidofovir [10]. A number of studies have been published on the development of HAdV quantitative PCR (qPCR) protocols [15]. We have previously developed a qPCR assay that can detect HAdV serotypes 11, 34, and 35 and shown that persistence of HAdV in the lower respiratory tract is common among immunocompromised hosts even without clinical adenoviral infections, and the viral load was correlated with low absolute lymphocyte counts [16]. In recent years a commercial qPCR assay kit was available for specific detection of HAdV which facilitates the diagnosis and monitoring of HAdV infections in clinical laboratories [17]. Adenovirus PCR is also included in a number of other commercial multiplex PCR systems for clinical diagnostics, and we have shown that some of the newer technologies such as the resequencing microarray could also be used for the diagnosis of gastroenteritis and conjunctivitis due to HAdV [18, 19].

In some of the PCR assays, the primers and probes were either species- or type-specific or there were base mismatches when compared with hexon gene sequences of some HAdV strains available in GenBank. Hence, we attempted to design a primer/probe set that can cover HAdV species A to G. We compared the performance of our in-house HAdV qPCR protocol with the commercial assay using archival clinical specimens and proficiency test samples.

## Results

Performance characteristics of the in-house laboratory-developed HAdV qPCR assay were evaluated with reference to the RealStar® Adenovirus PCR Kit. Analytical sensitivity (LoD) is the lowest concentration of HAdV DNA that can be detected in 95% of the replicates. For the in-house assay, the LoD for plasma was 2.06 log_10_ copies/mL (95% CI: 1.92–2.38 log_10_ copies/mL) and that for VTM was 2.31 log_10_ copies/mL (95% CI: 2.16–2.63 log_10_ copies/mL) (Table 1). For LLoQ determination, we have calculated the SD using the concentrations with all replicates shown positive for HAdV (Table 1). The LLoQ for plasma and VTM were 2.60 log_10_ copies/mL and 2.94 log_10_ copies/mL, respectively, based on the SD of no greater than 0.15 log_10_ copies/mL (Table 2), while the SD values of the replicates with the concentrations 2.30 log_10_ copies/mL for plasma, and 2.34 and 2.64 log_10_ copies/mL for VTM were greater than 0.15 log_10_ copies/mL (data not shown).

**Table 1:** PCR results used for the calculation of the analytical sensitivity

Sample matrix	Input concentration (log_10_ copies/mL)	Number of replicates	Number of positives	Hit rates (%)
Plasma	2.90	16	16	100
	2.60	16	16	100
	2.30	16	16	100
	2.00	16	15	93.75
	1.70	16	8	50
	1.40	16	3	18.75
	No plasmid DNA	16	0	0
Viral transport medium	2.94	16	16	100
	2.64	16	16	100
	2.34	16	16	100
	2.04	16	11	68.75
	1.74	16	5	31.25
	1.44	16	2	12.5
	No plasmid DNA	16	0	0

**Table 2:** Replication experiment to evaluate precision for plasma (A) and VTM (B)

Nominal (log_10_ copies/mL)	No. of sample tested	Mean HAdV DNA load (log_10_ copies/mL) Intra-assay	SD (log_10_ copies/mL) Intra-assay	%CV Intra-assay	Mean HAdV DNA load (log_10_ copies/ ml) Inter-assay	SD (log_10_ copies/mL) Inter-assay	%CV Inter-assay
A							
9	3	9.12	0.01	0.05	9.12	0.01	0.07
8	3	8.07	0.01	0.09	8.07	0.01	0.07
7	3	6.94	0.01	0.13	6.94	0.01	0.10
6	3	5.82	0.01	0.11	6.81	0.01	0.12
5	3	4.80	0.02	0.38	4.80	0.02	0.33
4	3	4.08	0.02	0.54	4.10	0.03	0.71
3	3	3.16	0.05	1.50	3.15	0.10	3.21
2.60	3	2.85	0.02	0.63	2.90	0.07	2.42
B							
9	3	9.11	0.01	0.05	9.12	0.02	0.17
8	3	8.03	0.01	0.10	8.02	0.01	0.17
7	3	6.93	0.01	0.22	6.92	0.02	0.26
6	3	5.87	0.01	0.19	5.88	0.02	0.26
5	3	4.91	0.02	0.37	4.91	0.01	0.25
4	3	4.05	0.05	1.27	4.05	0.04	1.00
3	3	3.10	0.06	1.84	3.10	0.04	1.44
2.94	3	2.92	0.02	0.57	2.92	0.06	2.11

The analytical specificity of the in-house HAdV qPCR assay was evaluated. The in-house assay did not show cross reaction with cytomegalovirus, Epstein-Barr Virus, varicella-zoster virus, herpes simplex virus types 1 and 2, human herpesvirus 6, 7, and 8, BK virus, JC virus, hepatitis B and C viruses, parvovirus B19, human bocavirus, respiratory syncytial virus, human metapneumovirus, human parainfluenza virus 1–4, human coronaviruses (229E, NL63, OC43, HKU1, SARS-, and MERS-CoV), human enterovirus and rhinovirus, human parechovirus, and influenza viruses A, B, and C.

**Fig. 1:**
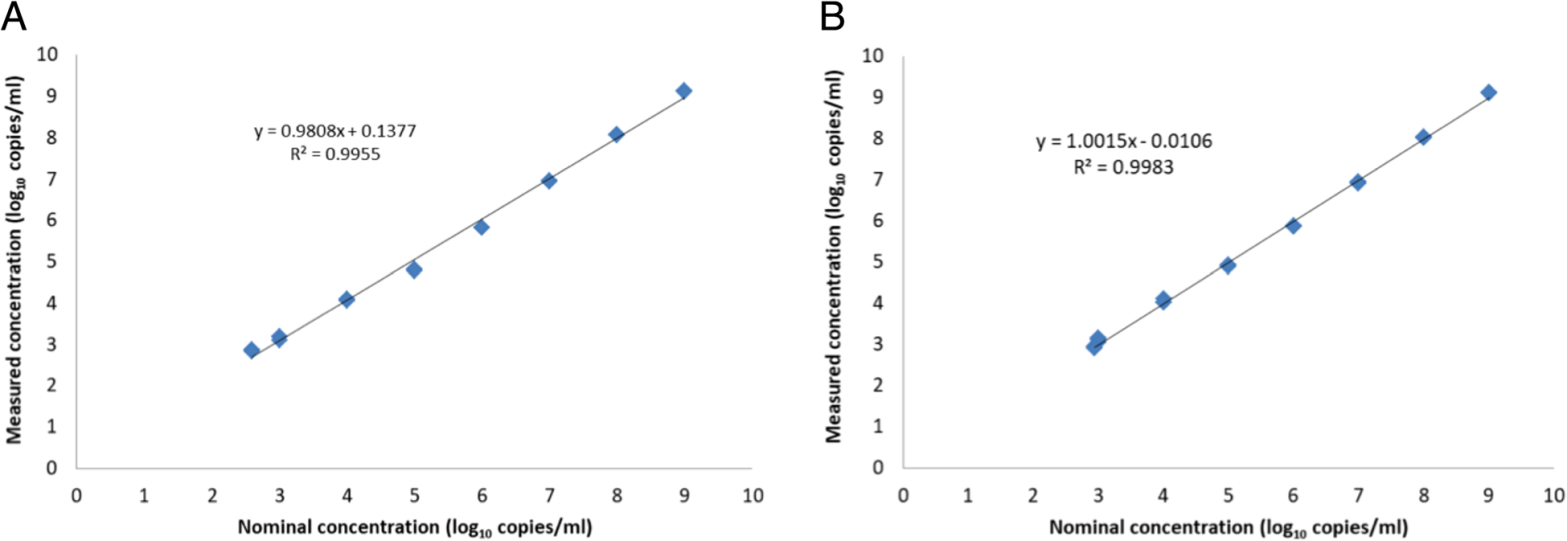
Linear range of the in-house laboratory-developed HAdV qPCR assay for plasma **a** and VTM **b**

Testing of the in-house HAdV qPCR assay across the range of detection from 2.60 to 9 log_10_ (plasma) and 2.94 to 9 log_10_ (VTM) copies/mL demonstrated excellent agreement between expected and observed viral loads with a coefficient of determination (R^2^) of 0.996 and 0.998, respectively (Fig. 1). In the replication experiment for evaluating the intra- and inter-assay variations, total imprecision (%CV) values for the 8 concentrations ranged from 0.07 to 3.21% for plasma and 0.17% to 2.11% for VTM (Table 2).

**Fig. 2:**
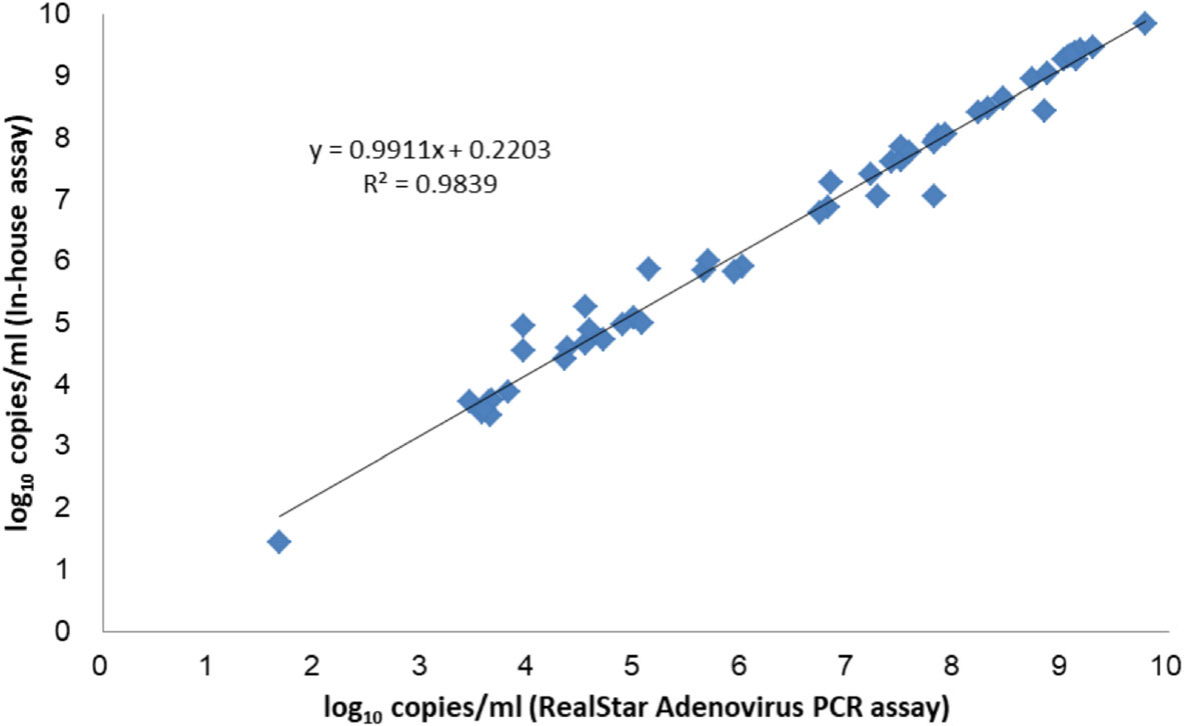
Correlation between HAdV viral loads determined by the in-house and commercial assays

**Fig. 3:**
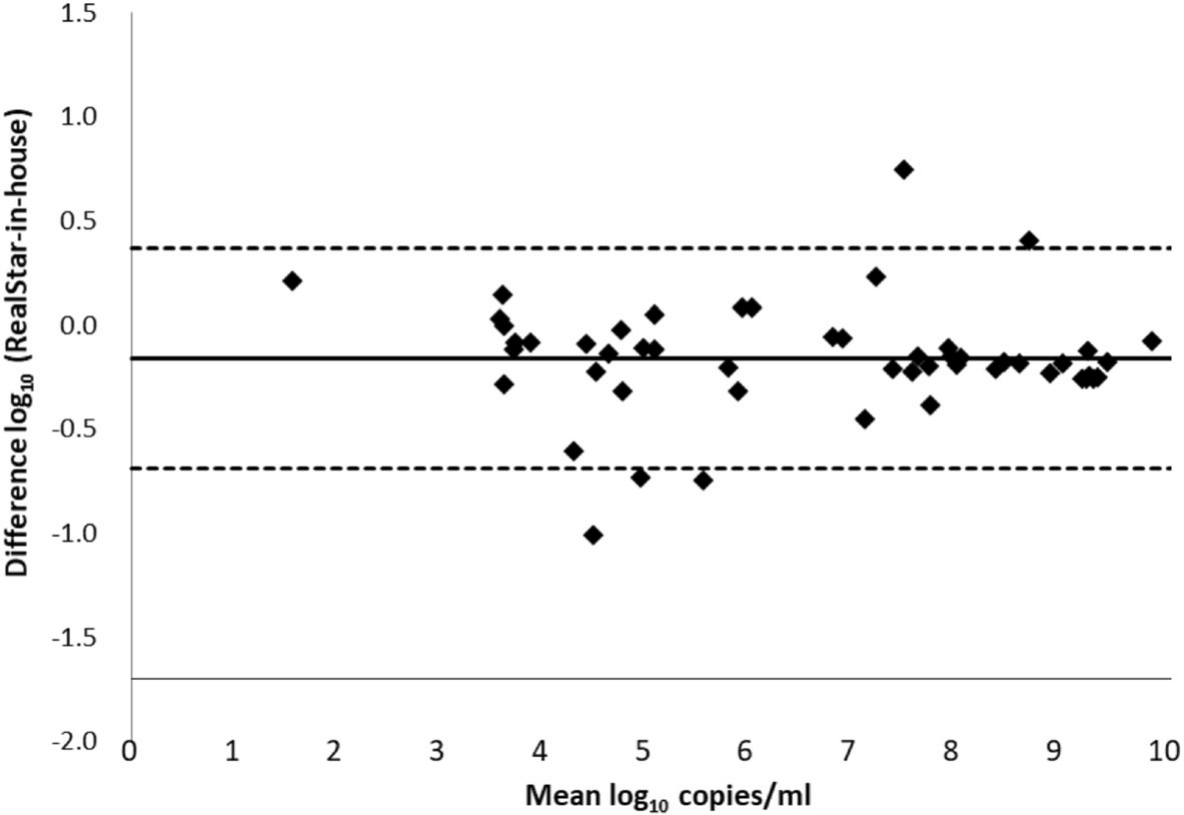
Bland-Altman plot of 52 samples with detectable viral loads tested by the in-house HAdV qPCR assay versus the RealStar® Adenovirus PCR Kit 1.0. The solid line represents the overall bias (mean difference) of the in-house assay, and the dotted lines represent the upper and lower limits of agreement (mean ± 1.96 SD)

The in-house HAdV qPCR assay was able to detect six adenovirus species A to F from the clinical and EQA samples. No species G was detected in this study. Among the 122 clinical specimens and 18 EQA samples subjected to HAdV DNA load quantification, 52 were positive (1 A, 26 B, 12 C, 1 D, 9 E, 2 F; the species were identified by BLASTn search from NCBI website using their partial hexon gene sequences) and 87 were negative by both in-house and commercial assays (see Additional file 1). No PCR inhibition was observed in any of the reactions for both assays. Using the RealStar® Adenovirus PCR Kit as the reference, the sensitivity and specificity of the in-house qPCR assay were 98.1% and 100%, respectively (Table 3). One sample that was not detected by the in-house assay but detected by the RealStar® Adenovirus PCR assay was excluded from linear regression analysis. There was a good agreement in the performance of the in-house HAdV qPCR assay compared to the RealStar® assay demonstrating a strong correlation with a coefficient of determination (R^2^) of 0.984 (Fig. 2). A Bland-Altman plot showed a mean difference of − 0.16 log_10_ copies/mL between the two assays (SD: 0.27) (Fig. 3).

**Table 3:** Comparison of the in-house HAdV qPCR assay and Realstar® Adenovirus PCR Kit 1.0

		Realstar® Adenovirus PCR kit 1.0		
		Positive	Negative	Total
In-house HAdV qPCR assay	Positive	52	0	52
	Negative	1	87	88
	Total	53	87	140

For the clinical sample (16 M466615) that was tested positive by the RealStar® Adenovirus PCR Kit but negative by our in-house assay, it was subjected to partial hexon gene PCR. The partial hexon gene could not be amplified for this sample. For another sample (16 M456964) that was tested positive by both in-house and commercial assays, its partial hexon gene could not be amplified, this was likely due to the low viral load in the sample (< 1.7 log_10_ copies/mL). For the EQA samples evaluation, the in-house HAdV qPCR assay could give the viral load values that are close to the consensus values in the reports of CAP and QCMD, and within our acceptance criteria for EQA samples (consensus value ±1.96 SD) (see Additional file 1). The data (i.e. the consensus results) on the samples from QCMD came from an External Quality Assessment study and was not part of a formal method comparison.

## Discussion

Nucleic acid amplification tests have become one of the most important laboratory diagnostic tools in clinical virology. In addition to the high sensitivity of the tests, the relative ease to quantify the viral loads, short turnaround time (as compared to conventional viral culture), and the ability to detect antiviral resistance genes and mutations all provide clinically relevant information for diagnosis and monitoring of patients with various viral infections. Commercial PCR kits have been widely available for the detection of many common viral pathogens; they offer the advantages of relative simplicity in testing procedures, well characterized performance by manufacturers, and in many cases, some degrees of automation and integration into the laboratory workflow. However, the availability of commercial kits remains limited for some of the less commonly encountered pathogens.

HAdV are well known to cause respiratory and enteric infections in the community. Outbreaks due to HAdV may involve individuals in institutions, including military recruits for which HAdV vaccines were specifically developed in the USA [20]. Severe respiratory tract involvement is an important complication in adenovirus infections, even in immunocompetent individuals [2]. Immunocompromised hosts, especially children, are at a high risk of developing severe and sometimes disseminated adenovirus infections with high mortality [3]. To the best of our knowledge, there are only two commercially available HAdV qPCR tests kits at the time of writing, the RealStar® Adenovirus PCR Kit (Altona Diagnostics) and Adenovirus R-Gene® (bioMérieux). In the current study, we compared our in-house laboratory-developed HAdV qPCR protocol with the RealStar® Adenovirus PCR Kit. The in-house protocol was adapted to the available molecular diagnostic facilities in our clinical laboratory.

Although a number of researchers have developed qPCR assays for the diagnosis and monitoring of adenovirus infections, many of the previous tests have intrinsic limitations in that some were only species-specific, while some so-called “pan-adenovirus” assays still showed base mismatches between the primers/probes and target gene sequences of HAdV available in GenBank (see Additional file 2). For example, one of the assays only included three serotypes (11, 34, and 35 of species B) [16]. In another study, the forward primer contained 1 base mismatch with species A, 2–3 mismatches with species B, 3 mismatches with species D, 2 mismatches with species E, 2 mismatches with species F, and 1 mismatch with species G; the reverse primer contained 1–3 mismatches with species A to F; and the probe contained 1–4 mismatches with species A to G [21]. One of the studies showed improved detection of HAdV species A to G by the use of several forward and reverse primers and probes that covered all species in a single reaction, but mismatches were still observed in the primers and probes [22]. In our study, only one primer/probe set was used using highly conserved primers and probe sequences. In our forward and reverse primer set, there was only 1 base mismatch at or near the 5′ end of the primer with some types of species B and F only. For the probe developed in this study, there was 1 base mismatch with F41, but this did not affect the detection because our in-house assay was able to detect two F41 strains from our clinical samples. In silico analysis also suggested that our primer and probe set should be able to detect species G, although we did not have species G strains in our collection to confirm this. Our in-house qPCR assay was shown to be 100% specific for HAdV with no cross-reactivity against 32 human viral pathogens. This compares favourably to the RealStar® Adenovirus PCR Kit which is also 100% specific when tested against 35 different viral and bacterial agents [23]. Unlike the commercial kit which has been evaluated for the seven species (A to G) of HAdV, our in-house assay has only been tested for species A to F as we did not have species G samples in our archive. The diagnostic performance of the in-house assay was evaluated using 122 clinical specimens and 18 EQA samples. The HAdV species was further confirmed by partial sequencing of the hexon gene. Our in-house assay achieved a high sensitivity (98.1%) when compared to the commercial kit, and there correlation of the two assays is very good (R^2^ = 0.984). Our in-house assay has a linear range up to 9 log_10_ copies/mL, which is comparable to other studies [21, 24, 25]. There were very low intra- and inter-assay variations as reflected in the %CV values.

At the time of writing, there was only one published study that evaluated the Altona RealStar® PCR assay against an in-house HAdV qPCR test in a reference centre [17]. The study also showed good correlation in the performance between the Altona RealStar® and in-house assays. However, only HAdV species A, B, C, D, and F were included in that study, with the majority of the strains being species B and C. The cost of PCR reagents for our in-house assay (US$1.4 per reaction) is much lower than that of the RealStar® Adenovirus PCR Kit (US$18 per reaction). Thus, our in-house assay may serve as a valuable tool for the low-cost and accurate detection and quantitation of HAdV DNA, while for the users who do not have expertise in the development of in-house assays (e.g. primer and probe design, recombinant plasmid preparation), the RealStar® Adenovirus PCR Kit would be an alternative for quantitative detection of HAdV in their laboratories because of its good diagnostic performance.

## Conclusions

We developed an in-house qPCR assay for the detection and quantitation of HAdV (species A to G). The performance of the assay correlated well with the commercially available Altona RealStar® Adenovirus PCR Kit with comparable sensitivity, specificity, and linear dynamic range.

## Methods

### Samples for evaluation

One hundred and twenty two archived clinical specimens submitted to the Department of Microbiology at Queen Mary Hospital between 2014 to 2017 were subjected to HAdV testing. These included 30 plasma, 1 serum, 4 cerebrospinal fluid (CSF), 46 respiratory specimens (including bronchoalveolar lavage, endotracheal aspirate, nasal swabs, nasopharyngeal swab and aspirate, throat swabs, tracheal aspirate, and sputum), 15 stool, 3 rectal swabs, 11 urine, 1 eye swab, 1 skin swab, and 10 biopsy specimens (see Additional file 1). For clinical validation, 18 samples including plasma and/or transport medium with various concentrations of HAdV or negative for HAdV from College of American Pathologists (CAP) and Quality Control for Molecular Diagnostics (QCMD) were used for external quality assessment (EQA). All samples were stored at − 70 °C prior to testing.

### Viral nucleic acid extraction

Plasma and serum (500 or 1000 μL), CSF (100 or 200 μL), respiratory, urine, and stool specimens and swabs (250 μL) were subjected to total nucleic acid (TNA) extraction by NucliSENS easyMAG extraction system (bioMérieux, Marcy-l’Étoile France), with the elution volumes of 25 μL, 50 μL, and 55 μL, respectively. Around 25 mg tissue sample (biopsy) was extracted by QIAamp DNA Mini Kit (Qiagen, Hilden, Germany), with the elution volume of 200 μL, according to manufacturer’s instructions.

### Sequence analysis of primers and probes for HAdV detection

In order to design a set of primers and probe that can detect all HAdV species, we performed a multiple sequence alignment of hexon genes of various types of HAdV species A to G using BioEdit version 7.2.5. The number of mismatches between the hexon gene sequences of HAdV strains available in GenBank and different primer/probe sets adopted from other studies were also identified [21, 22].

### Quantitative PCR

The primers and probes used for HAdV qPCR and monitoring PCR inhibition were shown in Table 4. A 20 μL reaction mixture contained 10 μL of 2× QuantiNova Probe PCR Master Mix (QIAGEN, Germany), 0.4 μM of each primer, 0.2 μM probe, and 5 μL sample template (TNA/DNA). The in-house qPCR assay was performed by LightCycler® 480 Instrument II (Roche, Rotkreuz, Switzerland). The PCR conditions consisted of 1 cycle at 95 °C for 2 min, followed by 50 cycles at 95 °C for 5 s and 60 °C for 30 s. The samples were run in parallel using RealStar® Adenovirus PCR Kit 1.0 (Altona Diagnostics GmbH, Hamburg, Germany), with the reaction volume of 30 μL containing 10 μL template according to manufacturer’s instructions. To monitor PCR inhibition in the in-house qPCR assay, internal control primers (0.4 μM each), probe (0.2 μM), and plasmid DNA with an 113 bp insert (1 μL with 1000 copies) that was used in our previous study [26] was added in each reaction.

**Table 4:** Primers and probes used in the present study

Target	Primer/probe sequence (5′–3′)		Tm (°C)	Amplicon size (bp)	PCR methodology
HAdV (hexon)	Forward	CAGTGGKCDTACATGCACATC	55.8	75	qPCR
	Reverse	GCGGGCRAAYTGCACSAG	60.3		
	Probe	FAM- CTCAGGTACTCCGARGC-MGB-NFQ	53.1		
Internal control	Forward	GTTCACCGATAGACCGCTG	55.5	113	qPCR
	Reverse	AAGAGCCCGGAATGTCAAGA	56.3		
	Probe	Cy5-ACTACCTGAGCACCCAGTCCGCCCT-BBQ	67.3		
HAdV (hexon)	Forward	GCCACCTTYTTCCCCATGGC	60.5	1004	Conventional PCR (1st round)
	Reverse	GTAGCGTTRCCGGCNGAGAA	59.8		
	Forward	TTCCCCATGGCNCACAACAC	59.6	956	Conventional PCR (nested); partial hexon gene sequencing
	Reverse	GCCTCRATGACGCCGCGGTG	65.2		

*bp* base pairs, *Tm* melting temperature

### Standards and controls

A plasmid standard was prepared using pCRII-TOPO vector (Invitrogen, Carlsbad, USA) cloned with a target insert (a 75 bp hexon gene of HAdV). The plasmid stock (2 × 10^10^ copies/μL) was diluted in AE buffer to prepare working stocks, which were aliquoted and stored at − 80 °C. The working stock was further diluted in AE buffer to final concentrations of 2 × 10^5^, 2 × 10^4^, 2 × 10^3^, 2 × 10^2^ and 2 × 10^1^ copies/μL as a quantification standard for the in-house qPCR assay. Plasmids with concentrations of 2 × 10^3^ and 2 × 10^1^ copies/μL were used as strong and weak positive controls, respectively. To monitor potential cross contamination during PCR setup, no-template control (PCR-grade water) was added into a well containing reaction mixture as a negative control.

### Adenovirus partial hexon gene sequencing

The HAdV-positive samples were subjected to partial hexon gene sequencing for species identification. Conventional PCR was performed using primers modified from a previous study [27] (Table 4). The PCR mixture (25 μL) contained template (2 μL of sample template for first-round PCR; 1 μL of first-round PCR product for nested PCR), 1× PCR buffer II, 2 mM MgCl_2_, 200 μM of each dNTP, 0.5 μM of each primer, and 1.25 U of Taq polymerase (Applied Biosystems, Foster City, USA). Both first-round and nested PCR were performed using an automated thermocycler (Applied Biosystems, Foster City, USA) with a hot start at 95 °C for 10 min, followed by 40 cycles of 94 °C for 1 min, 55 °C for 1 min, and 72 °C for 1.5 min and a final extension at 72 °C for 10 min. The PCR products were detected by agarose gel electrophoresis. Both strands of the PCR products were sequenced with an ABI 3130xl DNA Analyzer (Applied Biosystems, USA) using the PCR primers. HAdV species was determined by BLAST search from the NCBI website.

### Analysis of performance characteristics of the in-house test

The in-house HAdV qPCR assay was subjected to evaluation in terms of analytical sensitivity and specificity, linearity, precision, and accuracy. The analytical sensitivity (LoD) was identified by evaluating a dilution series of HAdV-negative plasma and viral transport medium (VTM) matrices spiked with recombinant plasmids with the concentrations from 2.90 to 1.40 log_10_ copies/mL (plasma) and from 2.94 to 1.44 log_10_ copies/mL (VTM) and then subjected to TNA extraction as described above (Table 1). The LLoQ was determined by two independent runs of each concentration tested in replicates. The plasmid DNA concentration in ng/μL was measured by BioDrop μLITE (BioDrop, UK), and double-stranded DNA copy number calculator (http://cels.uri.edu/gsc/cndna.html) was used to convert ng/μL into copies/μL. Definite amount of plasmid DNA was spiked into 1 mL of matrix (plasma/VTM), followed by doing 2-fold serial dilutions to obtain different concentrations for testing. Analytical specificity (cross-reactivity) was determined by testing genomic DNA/RNA extracted from other viruses causing similar symptoms to adenovirus infections. The linear range of the in-house qPCR assay was evaluated by analyzing a dilution series of HAdV-negative plasma and VTM matrices spiked with plasmids with the concentrations from LLoQ to 10^9^ copies/mL (in triplicates for each concentration). The intra-assay variation was evaluated by using HAdV-negative plasma and VTM matrices spiked with plasmids with 8 concentrations spanning the reportable range (LLoQ to 10^9^ copies/mL; in triplicates for each concentration) in a single run, while the inter-assay variation was evaluated by the samples with 8 concentrations from LLoQ to 10^9^ copies/mL (in triplicates for each concentration) in two independent experiments (Table 2). Coefficient of variation (CV = SD/mean × 100) was calculated for each concentration.

### Statistical analysis

Probit regression analysis to determine the 95% detection limit of the in-house HAdV qPCR assay was performed as described previously [28]. LLoQ was defined as the lowest dilution with a SD within 0.15 log_10_ copies/mL [29, 30]. The within-run and between-run precision of the in-house qPCR method for quantifying HAdV DNA was expressed by the coefficient of variation (CV). Agreement between the in-house qPCR assay and the RealStar® Adenovirus PCR Kit 1.0 was determined using a Bland-Altman plot of the samples positive by both in-house and commercial assays. Correlation of viral loads obtained from the in-house and commercial assays was performed by linear regression analysis. Statistical analysis was performed using IBM SPSS Statistics 24.

## Additional files

Additional file 1: Clinical specimen information and PCR results. (XLSX 18 kb)

Additional file 2: Multiple sequence alignment of hexon genes of human adenovirus species A to G and primer and probe sequences. (PDF 406 kb)

## Abbreviations

HAdV: Human adenoviruses
LLoQ: Lower limit of quantification
LoD: Lower limit of detection
qPCR: Quantitative polymerase chain reaction
VTM: Viral transport medium
