# Supplementary material for: Comparative evaluation of a laboratory-developed real-time PCR assay and RealStar® Adenovirus PCR Kit for quantitative detection of human adenovirus
Source: Virol J. 2018 Sep 27;15:149. doi: 10.1186/s12985-018-1059-7 (PMC6161464; doi:10.1186/s12985-018-1059-7)

Additional file 2: Multiple sequence alignment of hexon genes of human adenovirus species A to G and primer and probe sequences. Primer and probe sequences were adopted from (**A**) the present study, (**B**) Heim et al. and (**C**) Alsaleh et al.

(A) The present study

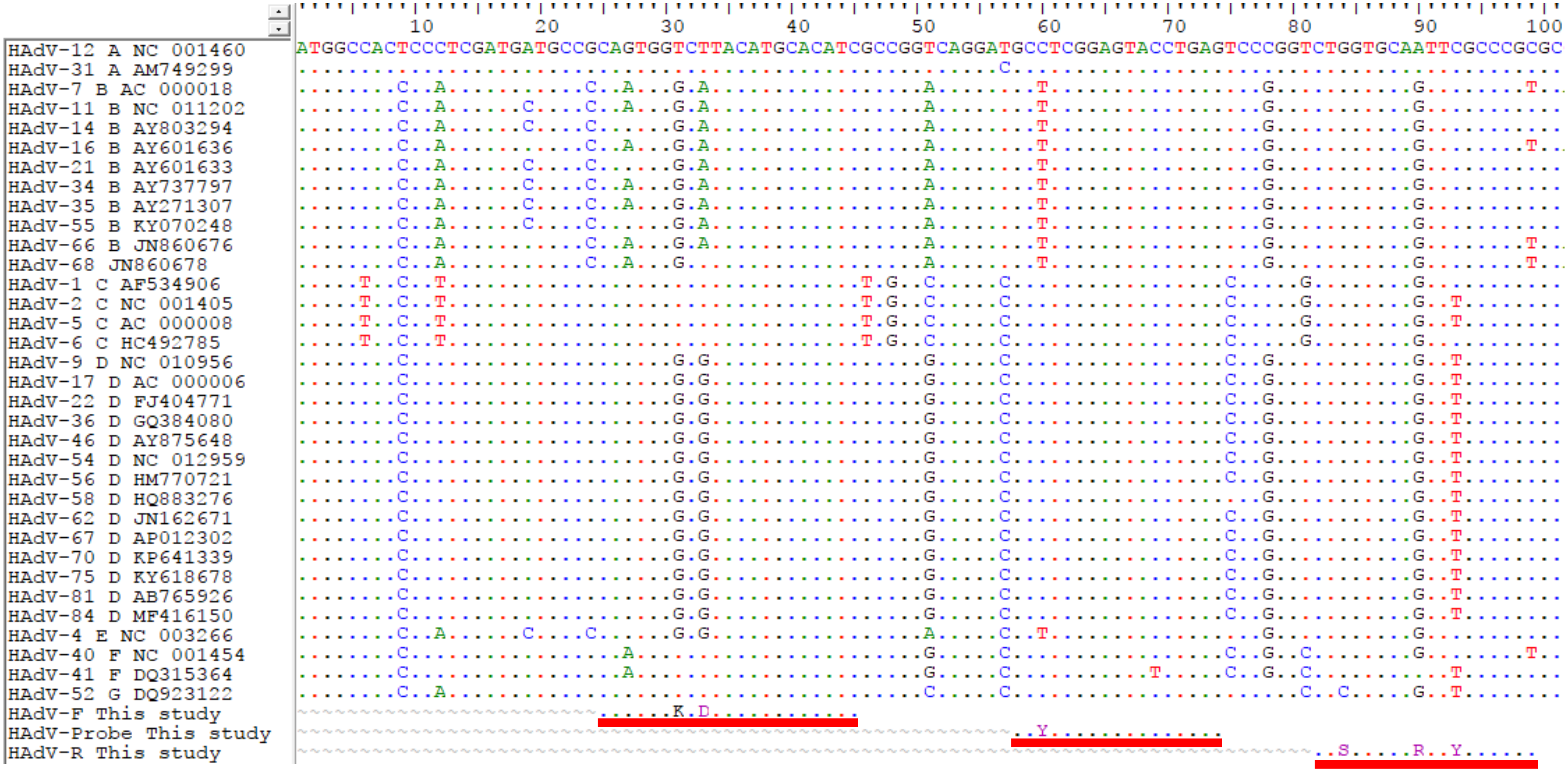

(B) Heim et al.

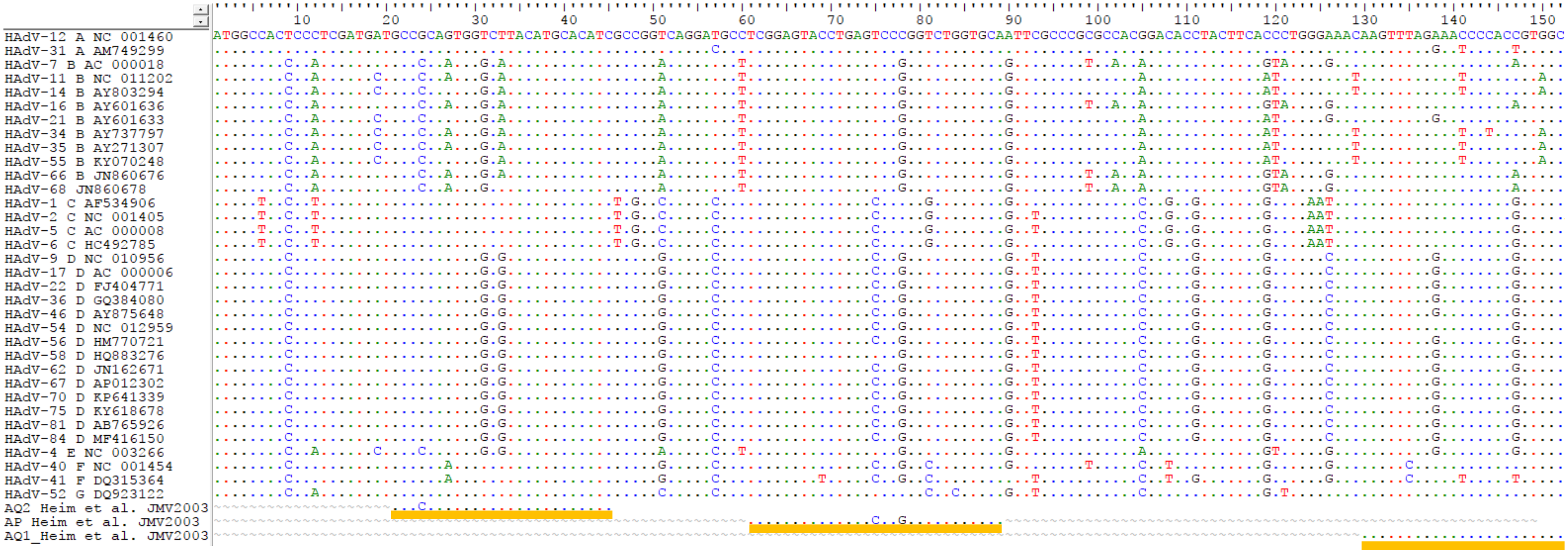
10 20 30 40 50 60 70 80 90 100 110 120 130 140 150
HAdV-12 A NC 001460
HAdV-31 A AM749299
HAdV-7 B AC 000018
HAdV-11 B NC 011202
HAdV-14 B AY803294
HAdV-16 B AY601636
HAdV-21 B AY601633
HAdV-34 B AY737797
HAdV-35 B AY271307
HAdV-55 B KY070248
HAdV-66 B JN860676
HAdV-68 JN860678
HAdV-1 C AF534906
HAdV-2 C NC 001405
HAdV-5 C AC 000008
HAdV-6 C HC492785
HAdV-9 D NC 010956
HAdV-17 D AC 000006
HAdV-22 D FJ404771
HAdV-36 D GQ384080
HAdV-46 D AY875648
HAdV-54 D NC 012959
HAdV-56 D HM770721
HAdV-58 D HQ883276
HAdV-62 D JN162671
HAdV-67 D AP012302
HAdV-70 D KP641339
HAdV-75 D KY618678
HAdV-81 D AB765926
HAdV-84 D MF416150
HAdV-4 E NC 003266
HAdV-40 F NC 001454
HAdV-41 F DQ315364
HAdV-52 G DQ923122
AQ2 Heim et al. JMV2003
AP Heim et al. JMV2003
AQ1_Heim et al. JMV2003

(C) Alsaleh et al. (Mod1-PCR)

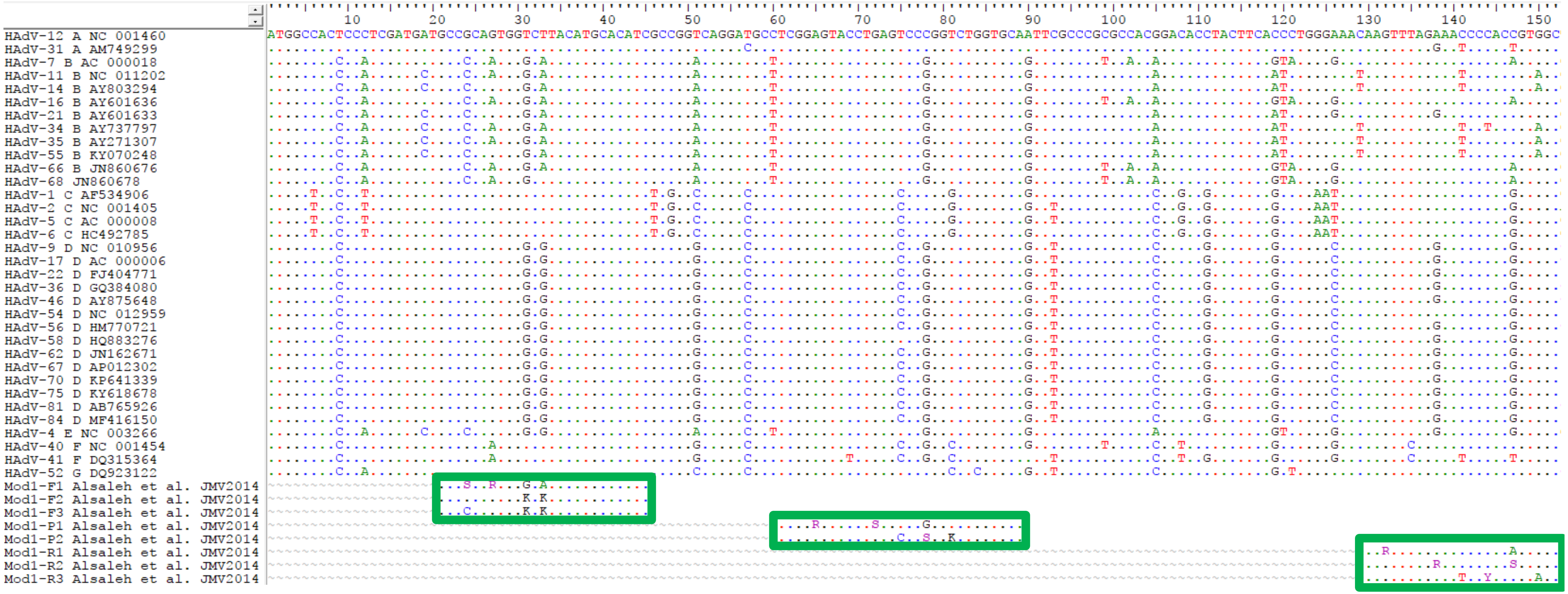

(C) Alsaleh et al. (Mod2-PCR)

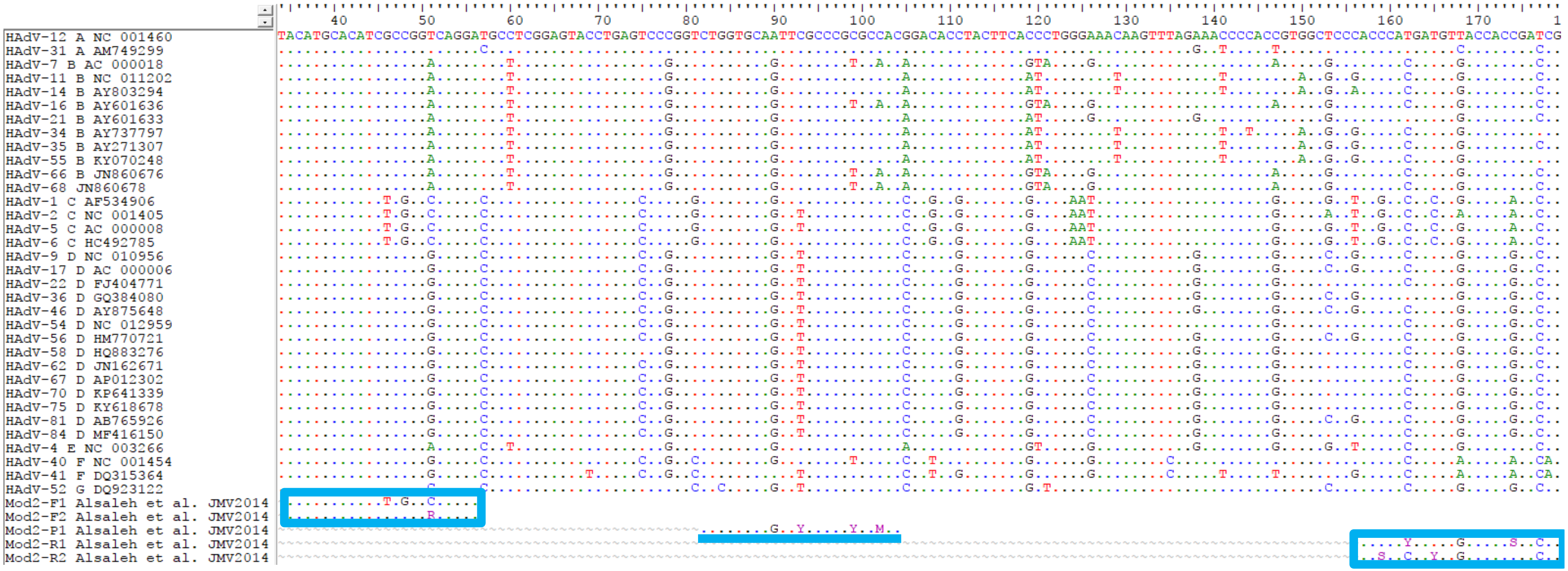

Supplement: Supplementary file 2 — Multiple sequence alignment of hexon genes of human adenovirus species A to G and primer and probe sequences. (PDF 406 kb) [file 12985_2018_1059_MOESM2_ESM.pdf]
